# In the Right Place at the Right Time: Regulation of Cell Metabolism by IP3R-Mediated Inter-Organelle Ca^2+^ Fluxes

**DOI:** 10.3389/fcell.2021.629522

**Published:** 2021-03-02

**Authors:** Ulises Ahumada-Castro, Galdo Bustos, Eduardo Silva-Pavez, Andrea Puebla-Huerta, Alenka Lovy, César Cárdenas

**Affiliations:** ^1^Geroscience Center for Brain Health and Metabolism, Center for Integrative Biology, Faculty of Sciences, Universidad Mayor, Santiago, Chile; ^2^Department of Neuroscience, Center for Neuroscience Research, Tufts University School of Medicine, Boston, MA, United States; ^3^Buck Institute for Research on Aging, Novato, CA, United States; ^4^Department of Chemistry and Biochemistry, University of California, Santa Barbara, Santa Barbara, CA, United States

**Keywords:** IP3Rs, calcium, endoplasmic reticulum, mitochondria, lysosome, metabolism, inositol triphosphate (IP3) receptors

## Abstract

In the last few years, metabolism has been shown to be controlled by cross-organelle communication. The relationship between the endoplasmic reticulum and mitochondria/lysosomes is the most studied; here, inositol 1,4,5-triphosphate (IP3) receptor (IP3R)-mediated calcium (Ca^2+^) release plays a central role. Recent evidence suggests that IP3R isoforms participate in synthesis and degradation pathways. This minireview will summarize the current findings in this area, emphasizing the critical role of Ca^2+^ communication on organelle function as well as catabolism and anabolism, particularly in cancer.

## Introduction

The life of a cell appears as an entanglement of complex networks of synthesis and degradation of molecules in a dynamic equilibrium essential for its viability (Muchowska et al., 2020). Within the cell, metabolic networks are completely intertwined, leading to the generation of special domains. Such domains contain a dynamic equilibrium of networks maintained by a series of regulators, particularly ions, such as calcium (Ca^2+^) (Bygrave, 1967).

Calcium is a regulator and cofactor of several fundamental cellular processes, including gene transcription, secretion, apoptosis, cell proliferation and differentiation, membrane excitability, motility, fertilization, autophagy, and metabolism (Berridge et al., 2000; Clapham, 2007). Inside the cell, Ca^2+^ is stored in several organelles, such as the endoplasmic reticulum (ER), mitochondria, nuclear envelope, Golgi apparatus, and lysosomes (Cárdenas et al., 2010a; La Rovere et al., 2016). The complex temporal and spatial control of Ca^2+^ communication between these organelles is achieved by several ion transporters and channels located in the membranes of these internal stores (Bootman and Bultynck, 2020). The main Ca^2+^ store of the cell is the ER (Berridge et al., 2003), which releases Ca^2+^ to the cytoplasm and other organelles, through ryanodine receptors (RyRs) (Lanner et al., 2010) and inositol 1,4,5-triphosphate (IP3) receptors (IP3Rs) (Mikoshiba, 2015). The RyRs display a rather limited tissue distribution, mainly in excitable cells such as neurons and myocytes (Shkryl, 2017), while IP3Rs are the most ubiquitous intracellular Ca^2+^ channels (Parys and De Smedt, 2012; Mak and Foskett, 2015).

Within organelles, Ca^2+^ plays additional roles regulating different aspects of organelle function, including metabolism. The regulatory role of Ca^2+^ on metabolic networks is supported by physical interactions between organelles that allow ion and molecule fluxes (Scorrano et al., 2019). These physical interactions have been defined as “membrane contact sites” (MCSs), which are highly dynamic interaction zones between two or more organelles characterized by close proximity between their membranes (10–80 nm), possessing specific functions and having a well-defined proteome and lipidome (Scorrano et al., 2019). So far, MCSs have been reported to be structural platforms for Ca^2+^ fluxes and to actively participate in intracellular signaling associated with autophagy, lipid metabolism, membrane dynamics, cellular stress response, organelle trafficking, and organelle biogenesis (Prinz et al., 2020).

Despite the above, knowledge about the regulation of metabolism by Ca^2+^ is still unknown in many aspects. The information about IP3R-mediated Ca^2+^ release in MCS and its possible implication is scarce. Thus, the role of IP3R in the MCS and how it may impact metabolism through mitochondria and lysosomes, along with its possible impact on cancer, will be reviewed.

## IP3R: Structure, Isoforms, and Functions

Inositol 1,4,5-triphosphate (IP3) receptor is a family of three ligand-gated channels: type 1 IP3R (IP3R1), type 2 IP3R (IP3R2), and type 3 IP3R (IP3R3) (Prole and Taylor, 2019), each about 300 kDa and encoded by separate genes with around 70% homology in their amino acid sequences (Mikoshiba, 2007). The isoforms have the property of assembling into homo- or heterotetramers to form functional channels (Monkawa et al., 1995). They localize to the ER, Golgi (Bootman and Bultynck, 2020), nuclear envelope, and nucleoplasmic reticulum (Cárdenas et al., 2010a). The structure of the IP3R monomer can be divided into three functionally different domains (Boehning et al., 2001), among which there are two domains of high homology between the isoforms: the N-terminal domain with the ligand-binding pocket, including a suppressor region in addition to the IP3-binding core, and the C-terminal domain containing a pore region of six transmembrane helices forming the Ca^2+^ channel (Mikoshiba, 2007; Serysheva, 2014). The suppressor domain reduces the affinity of the IP3R for IP3 (Iwai et al., 2007); nevertheless, it is necessary for channel opening in response to its ligand (Uchida et al., 2003). The central cytosolic regulatory domain exhibits more sequence variability between subtypes and contains interaction sites for many intracellular regulators and interacting proteins (Patterson et al., 2004). The gatekeeper domain (residues 2590–2749 of IP3R1), also called the C-terminal coupling domain, is fundamental for the activity of the channel, as mutations in the domain or its interaction with antibodies results in a non-functional Ca^2+^ channel (Nakade et al., 1991; Uchida et al., 2003). This domain also serves as a binding place for several regulatory proteins (Foskett et al., 2007).

Inositol 1,4,5-triphosphate is produced by hydrolysis of phosphatidylinositol 4,5-bisphosphate (PIP2) in the cell membrane through phospholipase C (PLC) in response to extracellular stimuli such as hormones, growth factors, and neurotransmitters that bind to G-protein-coupled receptors (Cattaneo et al., 2014), as well as to receptor tyrosine kinases (Nakamura and Fukami, 2017). Every subunit of IP3R must be bound to IP3 to trigger the opening of the channel (Alzayady et al., 2016).

The regulators associated to Ca^2+^ release mediated by IP3R include free Ca^2+^ concentration in the cytosol (mentioned here as cytoplasmic Ca^2+^), as well as in the ER (mentioned here as reticular Ca^2+^), ATP, thiol modification, and phosphorylation by protein kinases (Boehning et al., 2001; Parys and De Smedt, 2012). Additionally, many different proteins can interact with the IP3R affecting its function (Patterson et al., 2004) like the anti-apoptotic B-cell lymphoma-2 (BCL-2) (Chen et al., 2004), BCL-XL (White et al., 2005), the myeloid cell leukemia 1 (MCL-1) (Eckenrode et al., 2010), the pro-autophagic Beclin 1 (Vicencio et al., 2009), Ca^2+^-dependent phospholipid-binding protein Annexin A1 (ANXA1) (Vais et al., 2020), IRBIT (Bonneau et al., 2016), and the ER stress sensor IRE1α (Carreras-Sureda et al., 2019).

Despite IP3R isoforms sharing a high level of sequence homology, they exhibit different properties. For example, they differ in affinity for IP3, which was established as IP3R2 > IP3R1 > IP3R3 (Prole and Taylor, 2019). Their differential modulation by cytosolic Ca^2+^ (Tu et al., 2005), ATP (Wagner and Yule, 2012), binding proteins (Higo et al., 2005), and post-translational modifications (Szado et al., 2008) shapes IP3R behavior in a subtype-specific manner. Additional complexity to IP3R-mediated Ca^2+^ signaling arises from splice variants (Nakagawa et al., 1991) and the fact that most cell types express two or three different isoforms (Wojcikiewicz, 1995) that may assemble into heterotetramers (Monkawa et al., 1995; Chandrasekhar et al., 2016).

The different isoforms show tissue- and cell-specific expression patterns, with one isoform often being predominantly expressed (Vermassen et al., 2004). For example, IP3R1 has a broad tissue distribution but is especially abundant in the cerebellum (and most of the central nervous system), where it was initially purified and characterized (Furuichi et al., 1993). It is also expressed in vascular smooth muscle cells, thyroid, uterus, and lymphocytes (DeSouza et al., 2007; Narayanan et al., 2012). IP3R2 is expressed in cardiac muscle, as well as liver, kidney, and other epithelial tissues (Klar et al., 2014), while IP3R3 is expressed in endothelial cells, testis, endocrine and exocrine pancreas, spleen, gastrointestinal tract, and thymus (De Smedt et al., 1997).

## IP3R at the ER–Mitochondria MCS and Cellular Metabolism

**FIGURE 1:**
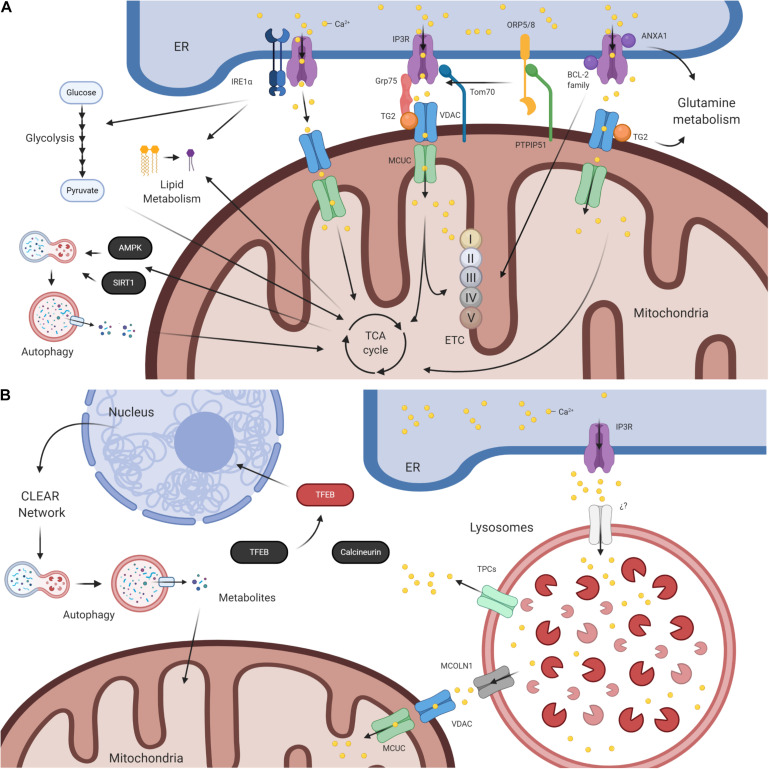
Effects in cellular metabolism of IP3R-mediated Ca^2+^ release in different membrane contact sites (MCS). **(A)** IP3R-mediated Ca^2+^ release in mitochondria–endoplasmic reticulum contact sites (MERCS) and its impact on cellular metabolism. Once Ca^2+^ is released from ER through the IP3R, it crosses the outer mitochondrial membrane (OMM) through VDAC and then the inner mitochondrial membrane (IMM) through MCU. TOM70, Grp75, and TG2 work as adaptors for IP3R–VDAC interaction. Additionally, IP3R interacts with proteins regulating Ca^2+^ release as IRE1a, BCL-2 family proteins, ANXA1, and ORP5/8. Once Ca^2+^ reaches the mitochondrial matrix, it regulates the TCA cycle and ETC complexes. Changes in both affect metabolic pathways as glycolysis, lipid metabolism, and autophagy (see details in the manuscript). **(B)** IP3R-mediated Ca^2+^ release from the ER passes through the lysosomal membrane using an unidentified channel (?). Lysosomal Ca^2+^ release regulates calcineurin activity, which regulates TFEB nuclear translocation. This works as a transcription factor to the CLEAR network, related to genes associated with autophagy and lysosomes. Additionally, Ca^2+^ release by the lysosomes through MCOLN1 crosses the OMM and the IMM using VDAC and MCU to reach the mitochondrial matrix, hypothetically regulating mitochondrial metabolism (see details in the manuscript). Figure created with BioRender.com.

The most characterized MCSs so far are the mitochondria–ER contact sites (MERCs) (Wu et al., 2018), which regulate many biological processes such as apoptosis, lipid synthesis, and early steps of autophagy, and several aspects of mitochondrial physiology, fusion–fission dynamics, biogenesis, and degradation (Gordaliza-Alaguero et al., 2019). The IP3R has been described as the most prevalent ER Ca^2+^ channel in MERCs. In those sites, the IP3R interacts with mitochondria through the voltage-dependent anion channels (VDACs) (Csordás et al., 2018). Additionally, it interacts with several adaptors such as the transglutaminase 2 (TG2) (D’Eletto et al., 2018), TOM70 (Filadi et al., 2018), and the glucose-regulated protein (Grp75) (Szabadkai et al., 2006; Honrath et al., 2017; Figure 1A). Finally, Ca^2+^ reaches the mitochondrial matrix through the channel mitochondrial calcium uniporter complex (MCUC), which interacts with many regulator proteins that affect its permeability to Ca^2+^ (De Stefani et al., 2016). The Ca^2+^ flux sustained by MERCs regulates mitochondrial metabolism and affects the activities of pyruvate dehydrogenase (PDH), isocitrate dehydrogenase (IDH), oxoglutarate dehydrogenase (OGDH), and electron transport chain (ETC) complexes III and V (Denton, 2009; Glancy and Balaban, 2012). If the ER–mitochondria Ca^2+^ flux is interrupted, p-AMPK levels increase, which, in turn, increases the activity of autophagy in a mTOR-independent manner (Cárdenas et al., 2010b, 2017). SIRT1 is also increased, which participates in the activation of autophagy (Cardenas et al., 2020; Figure 1A), and triggers mitochondrial fragmentation (Lovy et al., 2020). Autophagy is a process associated with degradative pathways of macromolecules, which generates monomeric metabolites that supply many metabolic pathways and in this manner maintain metabolic equilibrium and mitochondrial function (Rabinowitz and White, 2010; Guo et al., 2016; Ahumada-Castro et al., 2019). Additionally, the pro-autophagic protein Beclin-1 interacts with IP3R (Vicencio et al., 2009), and its lack of interaction induces autophagy, also contributing to regulating metabolism, but the mechanistic details are still unclear (Decuypere et al., 2011).

Furthermore, lipid accumulations associated with AMPK dephosphorylation have been observed in cellular models with inhibited MCU-dependent mitochondrial Ca^2+^ uptake. This is mediated by Ca^2+^-dependent protein phosphatase-4 (PP4) activity (Tomar et al., 2019). However, it remains mostly unclear to what extent lipid metabolism regulates IP3R-mediated Ca^2+^ fluxes or, *vice versa*, to what extent these Ca^2+^ fluxes regulate lipid metabolism via other intermediary proteins. Along these lines, proteins related to lipid transport have been described at the MERCS where they regulate Ca^2+^ flux between ER and mitochondria (Pulli et al., 2018). Moreover, a preprint from Giordano lab shows that oxysterol-binding protein-related proteins (ORP) 5 and 8 physically and functionally interact with MIB/MICOS complexes facilitating the transfer of phosphatidylserine (PS) from the ER to mitochondria (Rochin et al., 2019). In addition, ORP5 interacts with VDAC regulating the mitochondrial Ca^2+^ uptake at MERCS (Rochin et al., 2019; Figure 1A). Finally, ORP4L has been described extracting and presenting PIP2 to PLCβ3 (Zhong et al., 2019), facilitating IP3 generation. Also, ORP4L interaction with IP3R1 regulates its activity (Cao et al., 2019). Expectedly, all these interactions could cause mitochondrial bioenergetic alterations, involving additional metabolic pathways.

In all cases described above, it is not completely understood whether the IP3R isoforms contribute differently to this process. Recently, Bartok et al. (2019) clearly show that IP3R is necessary for ER–mitochondrial contact formation, and re-expression of each of the three isoforms alone was sufficient to restore the MERCs in triple IP3R-deficient (TKO) DT40 cells. Additionally, the authors show that IP3R2 turned out to be the most efficient isoform in delivering Ca^2+^ into mitochondria (Bartok et al., 2019). This result suggests that IP3R2 is essential for many aspects of cellular metabolism, but more studies need to be performed to clarify if this phenomenon observed in DT40 and HeLa can be extrapolated to other cell types and other physiological contexts.

B-cell lymphoma-2 protein family members, which also interact with the IP3R, have been shown to play a role in mitochondrial metabolism (Chong et al., 2020). In particular, BCL2 regulated oxidative phosphorylation (OXPHOS) at complex IV level (Chen and Pervaiz, 2010), BCL-XL regulated ATP synthase (Alavian et al., 2011), and MCL-1 regulated aspects of mitochondrial dynamics, mitochondrial physiology, and its metabolism (Perciavalle et al., 2012; Escudero et al., 2018; Figure 1A). In addition, TG2 and ANXA1 have been associated with glutamine metabolism (Figure 1A; Nurminskaya and Belkin, 2012) and regulation of reductive carboxylation, respectively (Rohwer et al., 2016). Whether these effects on metabolism are related to their interaction with IP3R and Ca^2+^ flux is unclear.

Finally, IRE1α, a protein related to ER homeostasis and its response to stress, particularly by the unfolded protein response (UPR) (Hetz et al., 2020), interacts with IP3R and plays a role in cellular metabolism (Figure 1A). Carreras-Sureda et al. (2019) described that IRE1α interacts with IP3R1, regulating IP3R-mediated Ca^2+^ release and its position in MERCS and in this manner regulates TCA cycle activity and MERCS. Although IRE1a has been described to regulate aspects of cellular metabolism such as lipid metabolism and glycolysis (Huang et al., 2019; Figure 1A), it is not clear whether this regulation is also mediated by IP3R-mediated Ca^2+^.

## IP3R at the ER–Lysosome MCS and Celullar Metabolism

In a similar way to MERCs, ER establishes membrane contact sites with lysosomes and endosomes, but these have not been completely characterized (Lee and Blackstone, 2020). A stable interaction between ER and early/late endosomes, probably related to the maturation process of these vesicles, has been shown (Friedman et al., 2011; Zajac et al., 2013). In addition, Ca^2+^ fluxes between these two organelles have been described. In fact, the ER is the most important source of lysosomal Ca^2+^, which can be delivered by all three IP3R isoforms (Atakpa et al., 2018; Figure 1B). This Ca^2+^ has been described as one of the counterions that the lysosome utilizes for its acidification (Christensen et al., 2002; Yang et al., 2019). Furthermore, changes in lysosomal pH generate a redistribution of lysosomes toward other areas of the ER with a lower density of IP3Rs, which finally interrupts this Ca^2+^ flow (Atakpa et al., 2018). Along with this, autophagy can be regulated by the release of lysosomal Ca^2+^ stores to cytoplasm, inducing the activity of calcineurin, a Ca^2+^-dependent phosphatase, which dephosphorylates the transcription factor EB (TFEB) and allows its translocation to the nucleus (Medina et al., 2015; Figure 1B). TFEB is a master regulator of the coordinated lysosomal expression and regulation (CLEAR) network, a gene network associated with the lysosome and autophagy (Palmieri et al., 2011). Another interesting fact described by Cang et al. (2013) pertains to the role of two pore channels (TPCs) on cellular metabolism. TPCs are endosome/lysosome-located ion channels selective to sodium (Na^+^) and Ca^2+^ that form a complex with mTOR. Upon starvation, when mTOR is inhibited, TPCs are actively transferring Ca^2+^ to the lysosome, which is necessary to maintain the lysosomal pH and amino acid efflux (Cang et al., 2013). Whether IP3R-mediated Ca^2+^ release to lysosomes plays a role in its degradative activity or in cellular metabolism is unknown. Interestingly, Peng et al. (2020) have described the occurrence of Ca^2+^ fluxes between lysosomes and mitochondria, via the lysosomal ion channel MCOLN1 and the mitochondrial transporter VDAC (Figure 1B). Such a discovery opened a new path to target Ca^2+^ regulation of mitochondrial metabolism for therapeutic purposes. Finally, cholesterol transfer between ER and lysosomes has been described, possibly having implications on low-density lipoprotein (LDL) metabolism (Höglinger et al., 2019). This transfer involves the cholesterol sensor oxysterol-binding protein-related protein 1L (ORP1L) and the integral ER proteins VAPA/B proteins (Höglinger et al., 2019). Certainly, the relationships between Ca^2+^ and lipids associated to lysosomes have been shown before (Van Der Kant and Neefjes, 2014); however, it is still unclear if IP3R Ca^2+^ release is involved.

## Implications in Cancer

For oncogenic processes, the rewiring of metabolic networks is an essential step (De Berardinis and Chandel, 2016). Many cancer hallmarks such as migration, invasion, excessive proliferation, apoptosis evasion, and angiogenesis, among others, are sustained by altered metabolism (Ward and Thompson, 2012). Alterations can be evidenced as changes in nutrient consumption (increase glutamine utilization) (Cluntun et al., 2017), changes in mitochondria to favor lipid and nucleotide synthesis to support growth (DeBerardinis et al., 2008), and generation of oncometabolites that favor proliferation and invasion of cancer cells (Sciacovelli and Frezza, 2016).

In all these instances, metabolic rewiring is coupled with modifications of Ca^2+^ fluxes, particularly those involving the ER Ca^2+^ store (Zhai et al., 2020). It has been described that pancreatic (Okeke et al., 2016), breast (Singh et al., 2017), colorectal (Pierro et al., 2014), glioblastoma (Kang et al., 2017), gastric (Sakakura et al., 2003), and lung cancers (Bergner et al., 2009) show increases in IP3R isoform levels, IP3R3 being the preponderant isoform (Rezuchova et al., 2019; Rosa et al., 2020), followed by IP3R2 (Vervloessem et al., 2014; Bartok et al., 2019). It could be speculated that these cells have a better mitochondrial metabolism fitness and lysosomal function that provide them with a proliferative and survival advantage.

Additionally, our group has described that cancer cells become “addicted to IP3R-mediated Ca^2+^ release,” which is essential for cancer cell survival (Cárdenas et al., 2017; Cardenas et al., 2020). In this context, it is interesting to note that many anti-apoptotic proteins, such as BCL2, BCL-XL, and MCL1, which are essential for the oncogenic processes, work as IP3R regulators. In addition, oncogenic proteins such as AKT, PML, and PTEN modify IP3R function. AKT, which is overactivated in many cancers, inhibits IP3R-mediated Ca^2+^ release (Marchi et al., 2012). Mutated PML (as is normally found in cancers) loses its ability to increase IP3R-mediated Ca^2+^ release (Missiroli et al., 2016). Finally, PTEN (Kuchay et al., 2017) increases IP3R-mediated Ca^2+^ release through its phosphatase activity. Currently, it is known and well accepted that IP3R-mediated Ca^2+^ fluxes in cancer are essential for cancer development, but whether specific changes in IP3R isoform patterns occur in different types of cancer has not been studied.

## Concluding Remarks/Perspectives

Cancer is a pathology like no other due to the presence of several layers of heterogeneity, which include different tissues and different oncogenic processes (Gerlinger et al., 2012; Venkatesan and Swanton, 2016). For these reasons, the search and development of new drugs capable to cure many types of cancers have been long and difficult. Cellular metabolism appears as a good target to tackle several types of cancer and its regulation by Ca^2+^ seems to be a good option to develop new drugs (Bustos et al., 2017). Regarding Ca^2+^, there is accumulated knowledge about the specific role of many ion channels in cancer cell physiology (Marchi et al., 2020). In this context, Xestospongin B (XeB), an alkaloid obtained from the marine sponge *Xestospongia exigua*, specifically inhibits IP3R-mediated Ca^2+^ release (Jaimovich et al., 2005), selectively inducing non-apoptotic cell death of some types of cancer cells (Cárdenas et al., 2017; Cardenas et al., 2020). In this case, Ca^2+^ flux interruption leads cancer cells into a metabolic and bioenergetic crisis that ends in cell death (Silva-Pavez et al., 2020). Other Xestospongins have been tested too, but they are not as selective as XeB (Gambardella et al., 2020). Similarly, the inhibition of the IP3 generation by ORP4L with the compound LYZ-81 has been shown to eradicate leukemia stem cells in a selective manner (Zhong et al., 2019). Another molecule tested to blunt the activity of the IP3R is the non-specific inhibitor, 2-aminoethoxydiphenyl borate (2APB), which induces apoptosis in gastric cancer cells through an unexplored mechanism (Sakakura et al., 2003). Along with the three cases mentioned above, there are other molecules that have been developed as IP3R inhibitors that need to be tested as potential anti-tumor drugs (Gambardella et al., 2020). On the other hand, several molecules and peptides have been tested and shown to activate IP3R-mediated Ca^2+^ release generating an overwhelming transfer of Ca^2+^ to mitochondria. These high Ca^2+^ concentrations induce apoptosis or sensitize cancer cells to chemotherapeutic drugs such as Bcl-2/IP3R Disruptor-2 (BIRD-2), which is a peptide (Bittremieux et al., 2019), and GGTi-2418, a geranylgeranyl transferase inhibitor of FBXL2 (Kuchay et al., 2017).

In all cases mentioned above, IP3Rs have emerged as essential Ca^2+^ channels for cancer cell survival. How different IP3R isoforms – homotetramers or heterotetramers – contribute to cancer development remains an area of research that requires more exploration. A better understanding of IP3R regulation and its effects on metabolism in normal and cancer cells will help to develop new therapeutic strategies to fight this devastating set of diseases in the future.

## Author Contributions

UA-C and CC designed and outlined the structure and contents of the review. UA-C, GB, ES-P, AP-H, AL, and CC contributed to the literature review, discussion, and writing of the manuscript. All authors contributed to manuscript revision and approved the version to be published.

## Conflict of Interest

The authors declare that the research was conducted in the absence of any commercial or financial relationships that could be construed as a potential conflict of interest.
